# *Aloe vera* gel confers therapeutic effect by reducing pyroptosis in ethanol-induced gastric ulcer rat model: Role of NLRP3/GSDMD signaling pathway

**DOI:** 10.1007/s11033-024-09329-4

**Published:** 2024-03-08

**Authors:** Amany O. Mohamed, Sary Kh. Abd-Elghaffar, Rehab A. Mousa, Amira A. Kamel

**Affiliations:** 1 Department of Medical Biochemistry and Molecular Biology, Faculty of Medicine, Assiut University, Assiut, Egypt; 2 Department of Pathology and Clinical Pathology, Faculty of Veterinary Medicine, Assiut University, Assiut, Egypt; 3 School of Veterinary Medicine, Badr University, Assiut, Egypt; 4 Department of Biochemistry, Faculty of Veterinary Medicine, Assiut University, Assiut, Egypt

**Keywords:** Gastric ulcer, *Aloe vera*, Pyroptosis, NLRP3, GSDMD

## Abstract

**Background:**

Gastric ulcer (GU) is a common gastrointestinal tract illness. *Aloe vera* has anti-inflammatory, antioxidant, and healing characteristics. This research sought to explore the therapeutic impact of *Aloe vera* gel on ethanol-provoked GU in rats and to elucidate the underlying mechanisms involved.

**Methods:**

An ethanol-induced GU rat model was constructed using forty male Wistar rats distributed at random into four groups: control, ulcer, pantoprazole, and *Aloe vera*. Gross evaluation of the stomach, ulcer index (UI), inhibition index, and gastric pH estimation were analyzed. Gastric malondialdehyde (MDA) and reduced glutathione (GSH) were determined using the spectrophotometric method, and serum gastrin level was measured by an enzyme-linked immunosorbent assay. Gastric nucleotide-binding domain, leucine-rich repeat, and pyrin domain PYD containing protein 3 (NLRP3) and gasdermin D (GSDMD) mRNA expression levels were estimated by quantitative real-time PCR. Finally, the histopathological examination of the glandular part of stomach tissue was done.

**Results:**

The ulcer group revealed a significant increase in MDA, gastrin, NLRP3, and GSDMD and a decrease in gastric pH and GSH compared to the control group. Gross investigations of the ulcer group revealed a hemorrhagic lesion in the stomach and an increase in UI. Also, histopathological results for this group showed severe epithelial loss, haemorrhage, inflammatory cell infiltration, and blood vessel congestion. However, *Aloe vera* treatment improved the gross, biochemical, molecular, and histopathological alterations induced by ethanol when compared to the ulcer group.

**Conclusions:**

*Aloe vera* exerted antiulcer activities through modulation of oxidant/antioxidant status, anti-secretory properties, and mitigation of pyroptosis.

## Introduction

Gastric ulcer is an erosion in the protective mucosal layer of the stomach that occurs when the gastric epithelial layer breaks down. It may be superficial or penetrates the submucosa and deep underlying tissue [1]. It is one of foremost common GIT diseases that influences about 10% of the people worldwide [2]. Now GU is described as the emerging “plague” of the twenty-first century [3]. GU is not a fatal disease, but it can lead to serious consequences like GIT bleeding, perforations, and penetration that have a serious effect on patient’s health and can be lethal [4].

It is a multifactorial disease that is associated with several factors, including *H. pylori* infection, nonsteroidal anti-inflammatory drugs, hyperacidity, alcohol intake, smoking, and stress [5]. Epigastric pain is a frequent symptom of GU and is worsened by meals. Hematemesis and melena may accompany it in extreme cases [6]. Disharmony between gastric aggressive factors [reactive oxygen species (ROS) and HCL] and defensive factors (mucous and antioxidants) is the primary pathophysiology of GU. As a result, the main therapeutic intervention for GU is to reduce gastric aggressive factors and increase defensive factors [7].

To date, the main strategy for GU treatment is inhibition of acid secretion and its neutralization by proton pump inhibitors, histamine 2 receptor antagonists, and antacids. These drugs have severe adverse effects such as osteoporosis, hypoacidity, gynecomastia, vitamin B12, and iron deficiency anemia [8]. Additionally, the FDA recalled ranitidine medications after finding elevated amounts of the cancer-causing contaminant N-nitrosodimethylamine (NDMA) [9]. So, finding natural, safe, and more effective treatment protocols with fewer side effects and more affordability has become a novel research hotspot nowadays. In this context, the usage of herbal remedies has gained interest and piqued researchers’ curiosity as a new therapeutic strategy in GU treatment [1].

*Aloe vera* is known as the “plant of immortality” and is one of the most prominent therapeutic plants used in folk medicine around the globe. Out of the 420 aloe species, it is the most bioactive one [10]. At least it contains about 75 biologically active ingredients such as vitamins, enzymes, minerals, sugars, steroids, hormones, saponins, amino acids, and phenolic compounds. It has anti-inflammatory, antioxidant, cytoprotective, immunomodulatory, wound healing, and anti-tumor effects [11]. According to these biological activities, *Aloe vera* can be used in GU healing.

Pyroptosis is a recently identified programmed cellular death process that is triggered by the activation of the NLRP3 inflammasome and mediated by GSDMD. It is characterized by cell expansion, plasma membrane pores creation, rupture, and proinflammatory mediators leakage, including interleukin 1 beta (IL-1β) and IL-18 into the outside of cells, amplifying the local or systemic inflammation [12]. Pyroptosis induces gastric mucosal cell death and pathological inflammation, which contribute to gastric epithelial impairment and the development of GU [13].

NLRP3 is one of nucleotide binding oligomerization domain (NOD)-like receptors (NLRs) family. They are cytoplasmic pattern recognition receptors that detect a wide range of stimuli and initiate immune responses and inflammation via the release of IL-1 family cytokines [14]. GSDMD is one of the most crucial members of the GSDM family, which executes pyroptosis, so pyroptosis is redefined as GSDMD-mediated cell death [15].

Gastrin hormone is a linear polypeptide hormone primarily produced in antral gastrin cells and, to some extent, in the duodenum. It stimulates acid production from the stomach’s parietal cells. The pathogenesis of GU is significantly influenced by increased gastrin production [16]. This study’s objectives were to assess *Aloe vera*’s therapeutic effect on oxidative stress, acidity of the stomach, and the pyroptotic pathway. This is in comparison with the standard drug pantoprazole, as well as to investigate its underlying mechanisms using an ethanol-induced GU rat model.

## Methods

### Experimental animals

The present study was done on 40 male Albino rats with body weights ranging from 150 to 200 g. Rats were purchased from the Animal House of the Faculty of Medicine, Assiut University. They were kept in metallic cages in healthy condition according to the study’s classification of groups. Prior to the experiment beginning, they were adapted to lab conditions for 2 weeks. They received an unlimited supply of commercial pellet food and water, as well as natural light and dark cycles. Following the instructions of Assiut University’s animal house, all experimental procedures were performed. The Assiut University Faculty of Medicine’s ethical committee gave its approval to the research protocol (IRB number 17300628).

### Chemicals

Absolute ethanol was obtained from Merck (CAS Number: 64-17-5), and pantoprazole was obtained from El-Esraa Pharmaceuticals (Batch No.: PPS/E-144/17). *Aloe vera* gel powder was purchased from Nature City (Boca Raton, Florida, USA). Analytical-grade chemicals and reagents were used.

### Experimental design

Forty rats were equally allocated into four groups (10 rats in each group).

1. Control group: The rats were given distilled water (D.W.) orally for 14 days [17].
2. Ulcer group: The rats were fasted for 24 h, then received one oral dosage of pure ethanol (0.5 ml/100 g) by gastric gavage. Rats were euthanised an hour following ethanol administration [18].
3. Pantoprazole group: The study’s reference medication was pantoprazole. 24 h post- GU induction, rats were given pantoprazole dissolved in D.W. (40 mg/kg/day) for 14 consecutive days via gastric gavage [19].
4. *Aloe vera* group: 24 h post-GU induction, rats were given *Aloe vera* gel dissolved in D.W. (200 mg/kg/day) for 14 consecutive days by gastric gavage [20].

On the 15th day, rats were euthanised by being cervically dislocated after chloroform inhalation anaesthesia.

### Blood samples collection

A rat’s retro-orbital sinus was used to take blood samples, which were collected in serum gel tubes. Serum was separated from blood samples by centrifuging them at 3000 rpm for 10 min at 4 °C. The supernatant was collected and kept at − 20 °C until the biochemical analysis of gastrin hormone.

### Collection of gastric content for measurement of gastric PH

Following the collection of blood samples, to gain access to the stomach, a median incision in the abdomen was made, and the stomach was excised from the gastroesophageal junction to the pyloric end. The stomach was cut across its greater curvature. After collection and centrifugation of gastric contents at 4 °C at 4000 rpm for 10 min, the supernatant pHwas measured using a digital pH meter.

### Estimation of ulcer index (UI) and calculation of percentage inhibition index (I%)

The excised stomach mucosa was gently washed using cold phosphate-buffered saline (PBS) (pH 7.4), spread on paraffin-coated petri dishes, and fixed with small pins with the mucosal surface directed upwards to allow better examination of lesions in the glandular part. An impartial person who was not aware of the experiment’s protocol assessed the ulcer index (UI) score macroscopically.

The criteria for assessment of UI were mucosal color, hyperemia, bleeding region, and ulcers number. These indices were scored in accordance with Singh et al. [21] as displayed in (Table 1). Additionally, the percentage inhibition index (I%), which was related to UI, was determined using the subsequent equation [22].

**Table 1:** The assessed score of ulcer index according to Singh et al. (2008)

Evaluation index	Score
No lesions (normal stomach)	0
Hyperemia	0.5–1
Hemorrhagic spots	1–2
1–5 small ulcers	2–3
Several small ulcers	3–4
1–5 small and 1–3 large ulcers	4–5
Several small and large ulcers	5–6
Stomach full of ulcers or perforations	6

$$\mathrm{Inhibition index }(\mathrm{I\%}) =\frac{(\mathrm{UI ulcerated group }-\mathrm{ UI treated group})}{\mathrm{UI ulcerated group }}\times 100$$

### Tissue samples

After the UI measurement, the stomach was divided into three pieces. One piece was liquid nitrogen snap frozen and kept at − 80 °C for qRT-PCR. The second piece was liquid nitrogen snap frozen and then held at − 80 °C to create 10% tissue homogenate for MDA and GSH spectrophotometric measurement. The third piece was fixed in 10%formalin for histological examination.

### Measurement of the gastric level of oxidant and antioxidant biomarkers (MDA and GSH)

For the determination of gastric tissue MDA and GSH, 0.1 g of gastric tissue was homogenized in 0.9 mLs of cold PBS (pH 7.4) to yield 10% tissue homogenates. Next, the centrifugation of homogenates was done for 15 min at 4000 rpm and 4 °C. Then the supernatants were used to determine MDA and GSH by spectrophotometric method using commercially available kits (Catalogue Nos. MD 25 29 and GR 25 11, respectively) provided by Biodiagnostics, Egypt, and in accordance with the instructions given by the manufacturer as mentioned by Kei [23] and Beutler et al. [24]. The method provided by Lowry et al. was used to estimate total protein [25]. MDA and GSH levels were normalized to protein content in each sample and represented as nmole/mg protein and mmole/mg protein, respectively.

### Serum gastrin assay

Serum gastrin hormone was quantified using the ELISA technique by a commercially available ELISA kit (catalogue No. 201-11-0967 m, Sun Red Biotech Co., Ltd., China) according to the producer’s directions. Serum gastrin concentration is measured in pg/ml.

## Quantitative real time-polymerase chain reaction (qRT-PCR)

### RNA extraction

The RNeasy Mini Kit (catalogue No. 74104, Qiagen, Germany) was used in order to extract total RNA from stomach tissue as directed by the producers. The acquired RNA concentration and purity were then assessed at 260 nm and 280 nm using a Nanodrop® (Epoch Microplate Spectrophotometer, Biotek, VA, United States) and maintained at − 80 °C until the following stage in the Medical Research Centre, Assiut University, Assiut.

#### Complementary DNA (cDNA) synthesis

Extracted gastric RNA was reverse transcribed into cDNA in accordance with the producer’s instructions using a High-Capacity cDNA Reverse Transcription Kit with RNase Inhibitor (catalog No. 4374966, Thermo-Fisher Scientific, USA). The recovered cDNA was subsequently diluted 1:5 by PCR-grade water and kept at − 20 °C for the following step.

#### Quantitative real-time PCR

The Maxima SYBR Green qPCR master mix (2) kit (Catalogue No. K0251, Thermo-Fisher Scientific, USA) was used to perform qRT-PCR. To determine the relative mRNA expression of NLRP3 and GSDMD. The generated cDNA was utilized as a template for amplification in a 25 µl PCR reaction volume using the specific primers for the genes. This was done using Applied Biosystems 7500 Fast Real-time PCR equipment (Germany). The PCR cycling conditions were as follows: the initial denaturation stage at 95 °C for 10 min, followed by 40 cycles of 95 °C for 15 s and 60 °C for 60 s. As an internal control in the same sample, the expression of a housekeeping gene (β-actin) mRNA was used for normalization of the target genes mRNA expression. The cycle threshold (Ct) values for target genes and the housekeeping gene were estimated. The relative expression of target genes was calculated using the 2^−ΔΔCT^ formula [26].

Primers were checked for particularity using the Primer-Blast program from the National Centre for Biotechnology Information [https://www.ncbi.nlm.nih.gov/tools/primer-blast/]. The primers were obtained from Invitrogen, UK. They were reconstructed following the producer’s directions. Primer sequences of NLRP3, GSDMD, and β-actin are shown in Table 2.

**Table 2:** Primer sequences of rat NLRP3, GSDMD, and β-actin genes

Gene	Primer sequence	Accession number	Product length
NLRP3	Forward: 5′ CAGACCTCCAAGACCACGACTG 3′ Reverse: 5′ CATCCGCAGCCAATGAACAGAG 3′	NM_001191642.1	127
GSDMD	Forward: 5′ CCAACATCTCAGGGCCCCAT 3′ Reverse: 5′ TGGCAAGTTTCTGCCCTGGA 3′	NM_001400994.1	139
β-actin	Forward: 5′ TGTCACCAACTGGGACGATA 3′ Reverse: 5′ GGGGTGTTGAAGGTCTCAAA 3′	NM_031144.3	165

#### Histopathological examinations

Excised gastric samples were fixed with 10% formalin for one day, dried using ethanol ascending grades, washed in xylol, and then impregnated in soft paraffin for two hours at 50 °C before being inserted in hard paraffin blocks. Tissue sections with a 5 µm thickness were cut, mounted on glass slides, and stained with hematoxylin and eosin (H & E) to identify the histopathological changes using light microscopy [27].

#### Statistical analysis

The data analysis was done using GraphPad Prism version 7 (GraphPad Software Inc., San Diego, USA). The results were expressed as a mean ± SD. The data’s normal distribution was checked using the Shapiro–Wilk normality test. To determine statistically significant differences among groups, a one-way analysis of variance (ANOVA) test was used, and least significant difference (LSD) post hoc test was applied for multiple comparisons between different groups. P value < 0.05 was considered statistically significant.

## Results

### Gastric PH

**Fig. 1:**
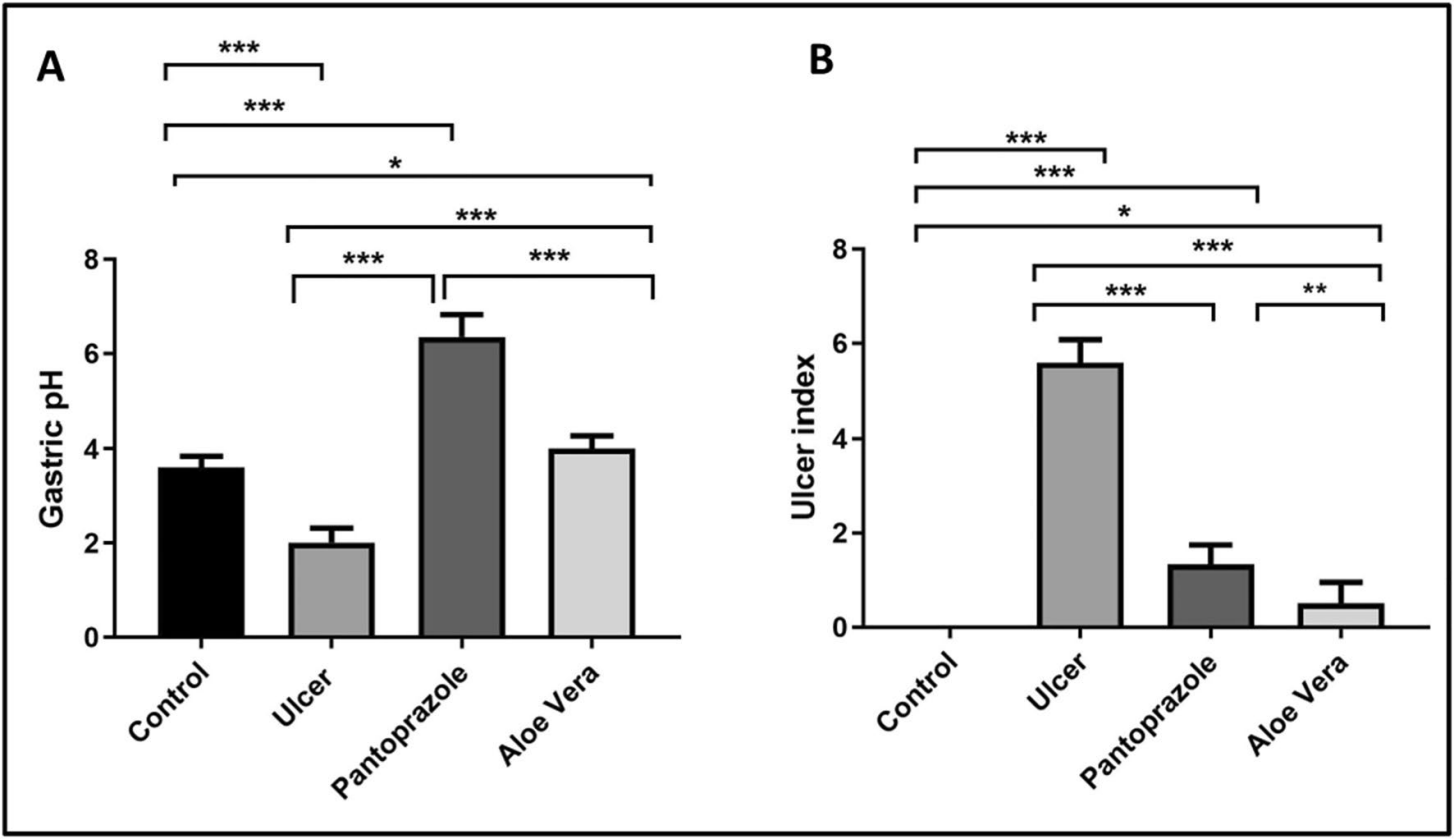
Gastric pH (**A**), ulcer index (**B**) in different groups. (n = 10; n is number/group). Data are presented as means ± SD. * p < 0.05; **, p < 0.01; ***, p < 0.001. using one-way analysis of variance with LSD post hoc test

This study revealed that gastric pH was significantly decreased in the ulcer group compared to the control group (P < 0.001). Conversely, treatment with Pantoprazole and *Aloe vera* showed a significant increase in gastric pH as compared to the ulcer group (P < 0.001 for each). Gastric PH was significantly elevated in the pantoprazole group when compared to the *Aloe vera* group (P < 0.001) (Fig. 1A).

### Gastric mucosa gross picture, ulcer index (UI), and inhibition index percentage (I%)

**Fig. 2:**
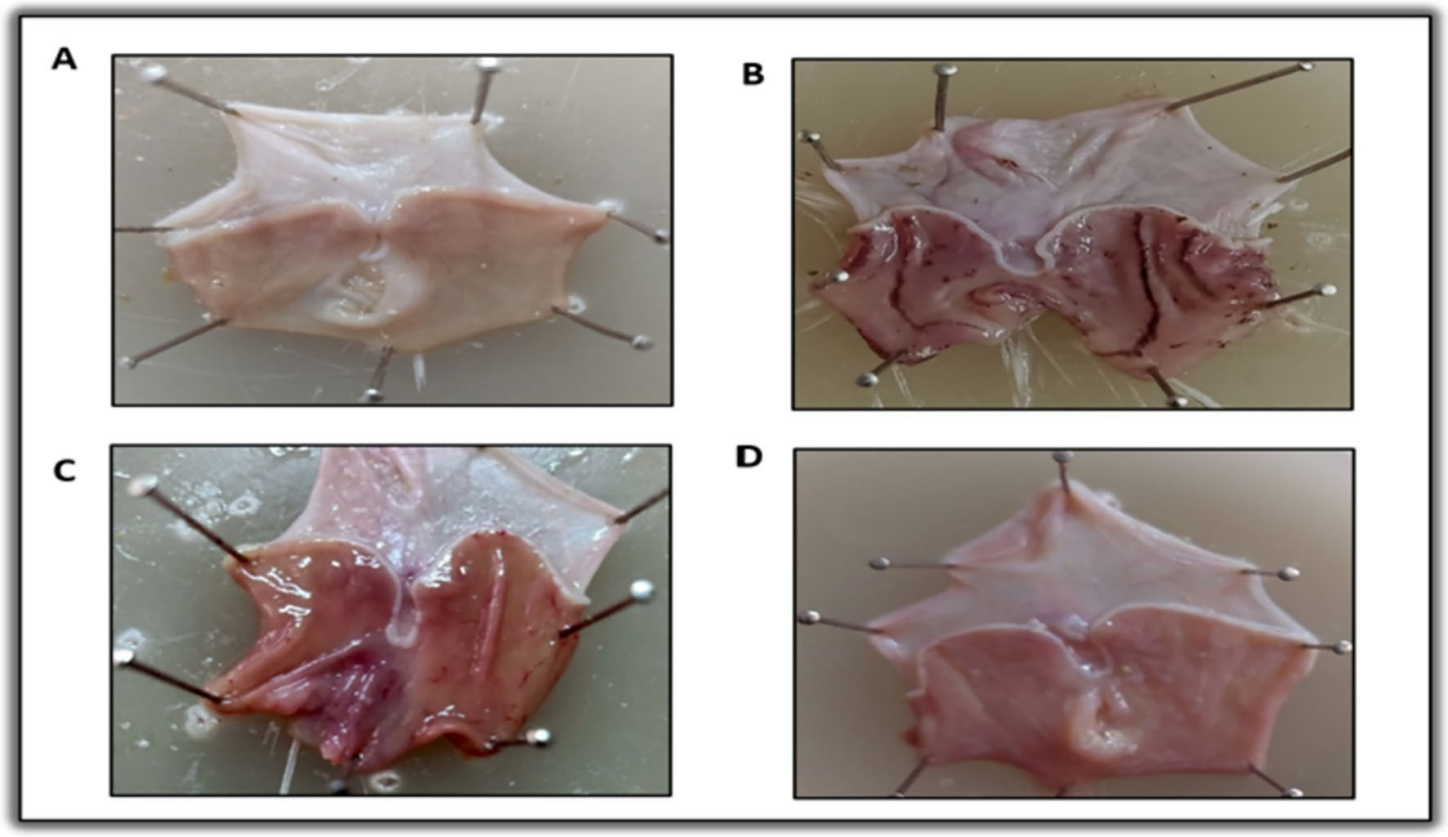
Gross picture of gastric mucosa in all experimental groups: control group (**A**), ulcer group (**B**), pantoprazole group (**C**), and *Aloe vera* group (**D**)

Macroscopicexaminations of the stomach mucosa revealed that the control group’s stomach had a normal mucosa without any apparent injuries visible to the naked eye (Fig. 2A). Conversely, the ulcer group displayed extensive hemorrhagic ulcerative lesions along the stomach mucosa (Fig. 2B). These lesions were quantified by a significantly increased UI compared to the control group (P > 0.001; Fig. 1B). Treatment with Pantoprazole revealed superficial hyperemia and minor hemorrhagic injuries (Fig. 2C), as well as a significantly lower UI (P > 0.001) than in the ulcer group (Fig. 1B). While *Aloe vera* treatment effectively healed the damaged mucosal layer, as no macroscopic lesions were detected in this group, only mild hyperemia was observed (Fig. 2D), proven by significantly lower UI values compared to the ulcer group (P > 0.001) (Fig. 1B). *Aloe vera* was found to significantly decrease UI when compared to the pantoprazole group (P = 0.001) (Fig. 1B). Calculating I% revealed that rats treated with *Aloe vera* had a net I% of 91% while rats treated with Pantoprazole had a net I% of 76.17%, indicating that *Aloe vera* had a greater therapeutic impact. UI and I% were summarized in Table 3.

**Table 3:** Effect of *Aloe vera* on ulcer index (UI) values and percentage inhibition index (I%)

	Ulcer Index (UI)	Inhibition index (I%)
Control group	NA	NA
Ulcer group	5.583 ± 0.37	NA
Pantoprazole group	1.333 ± 0.4^***^	76.17%
*Aloe vera* group	0.5 ± 0.3^***^	91%

UI values were expressed as mean ± SD and percentage inhibition index (I%)

P > 0.001 considered statistically significant compared to ulcer group

*NA* Not applicable

### Oxidant and antioxidant biomarkers (MDA and GSH)

**Fig. 3:**
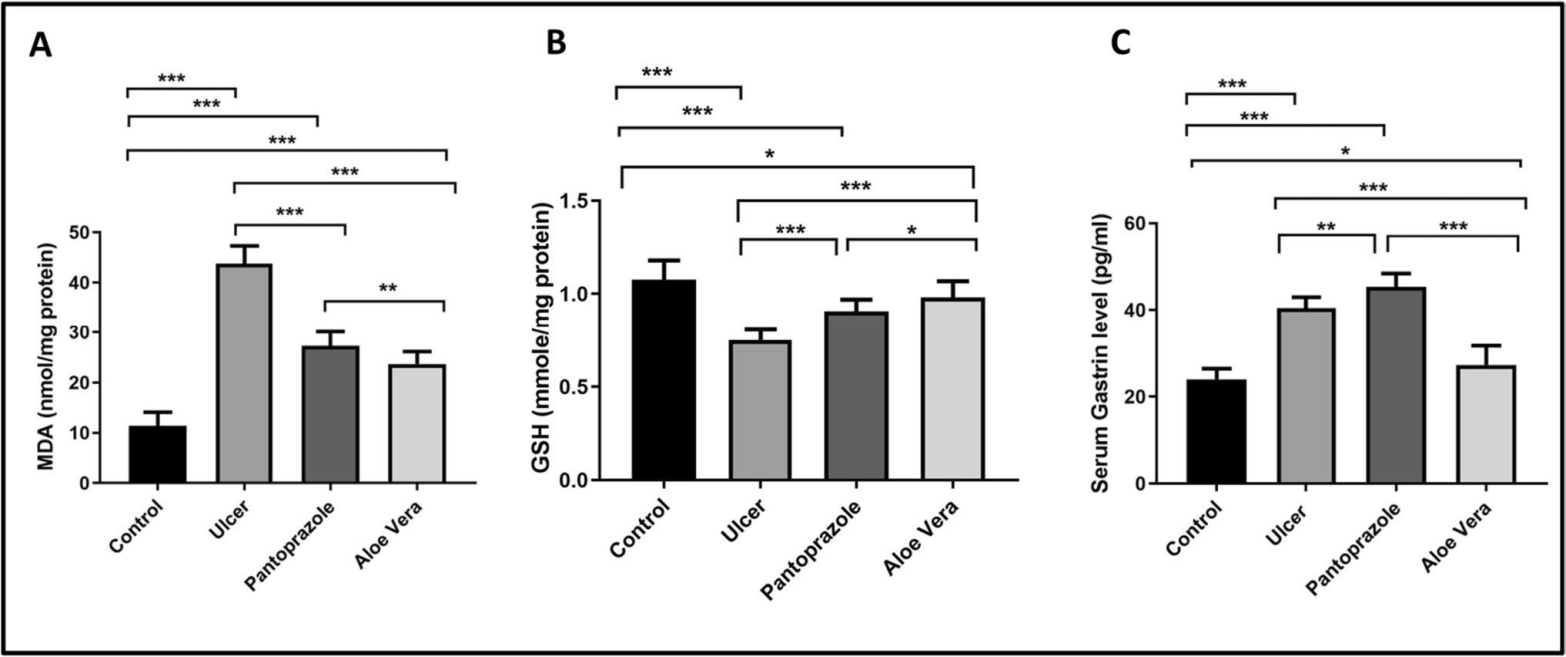
Levels of gastric MDA (**A**), gastric GSH (**B**), and serum gastrin (**C**) in different groups. Data are presented as means ± SD. * p < 0.05, ** p < 0.01, ***, p < 0.001 using the one-way analysis of variance with LSD post hoc test

The current study made it clear that the ulcer group had considerably greater stomach MDA levels than the control group (P < 0.001). Conversely, pantoprazole and *Aloe vera* treatments significantly decreased gastric MDA levels in comparison to the ulcer group (P < 0.001 for each). When compared to the Pantoprazole group, the *Aloe vera* group’s gastric MDA level was considerably reduced (P = 0.008; Fig. 3A).

Regarding GSH levels, we found that the level was substantially lower in the ulcer group compared to the control group (P < 0.001), while it was significantly higher in the pantoprazole group and the *Aloe vera* group when compared to the ulcer group. When compared to the Pantoprazole group, the gastric GSH level in the *Aloe vera* group was significantly higher (P = 0.039; Fig. 3B).

### Serum gastrin level

Serum gastrin levels were markedly higher in the ulcer group compared to the control group (P < 0.001). The administration of pantoprazole resulted in a significant increase in serum gastrin levels relative to the ulcer group (p = 0.002). In contrast, *Aloe vera* administration resulted in a significant decrease in its concentration compared to the ulcer group (P < 0.001). In comparison with the pantoprazole group, *Aloe vera* significantly decreased serum gastrin levels (p < 0.001; Fig. 3C).

### NLRP3 and GSDMD gene expression

**Fig. 4:**
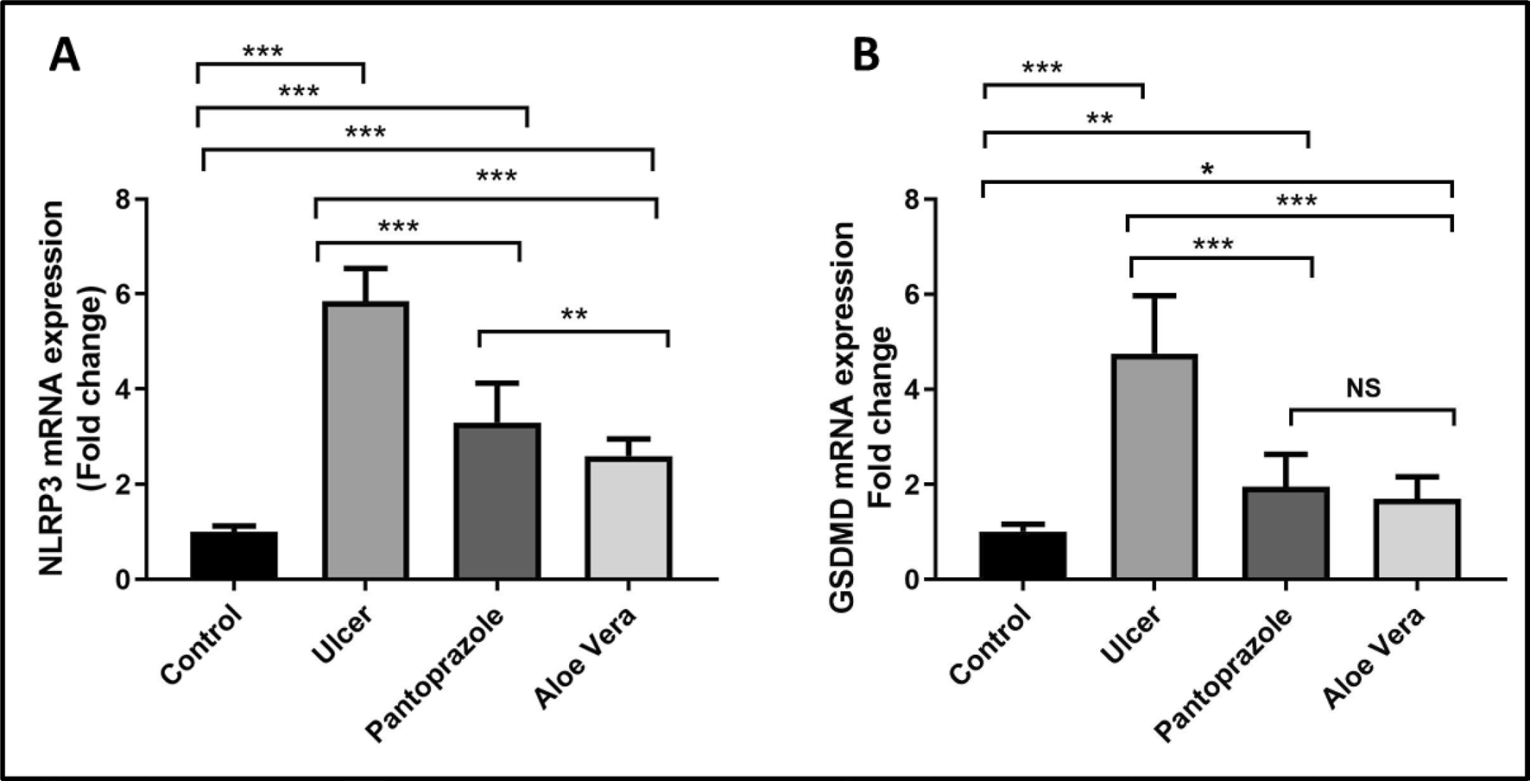
Relative mRNA expression (2^−ΔΔCt^) of gastric NLRP3 (**A**) and GSDMD (**B**). (n = 10; n is number/group). Graph represents mean ± SD (error bars). N.S P < 0.05, *P < 0.05, **P < 0.01, ***P < 0.001, NS: non-significant using the One-way analysis of variance with LSD post hoc test

This research elucidated that the relative mRNA expression of NLRP3 and GSDMD in stomach tissue had a significant upregulation in the ulcer group compared to the control group (P < 0.001 for each). Conversely, their expression levels had a significant downregulation in Pantoprazole group (P < 0.001) and the *Aloe vera* group (P < 0.001) compared to the ulcer group. In addition, NLRP3 expression level was significantly downregulated in the *Aloe vera* group compared to the pantoprazole group (P = 0.009; Fig. 4A). As regards GSDMD mRNA expression, there is no significant difference between the pantoprazole and *Aloe vera* groups (P = 0.428; Fig. 4B).

### Histopathological results

**Fig. 5:**
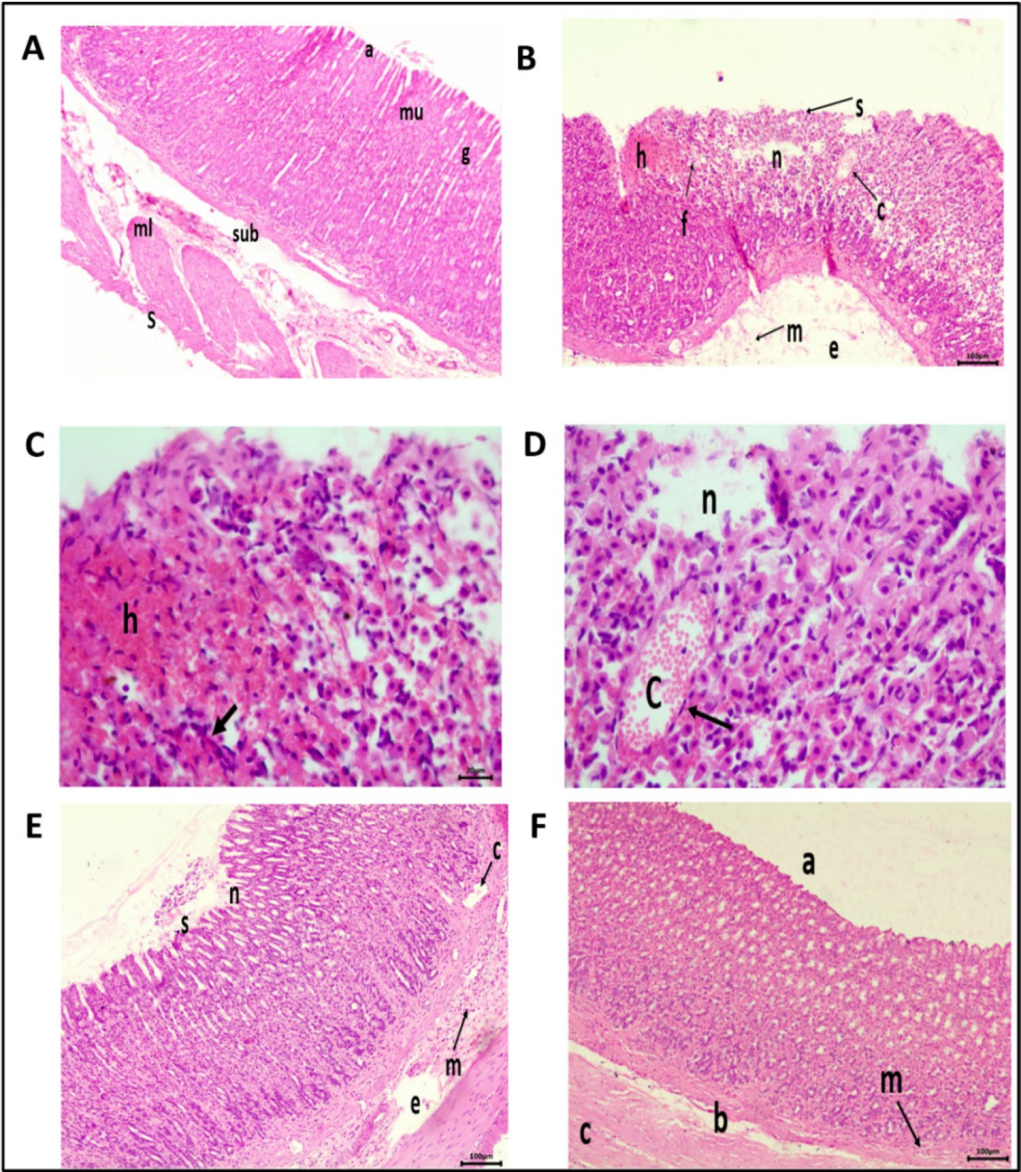
Photomicrographs of stomach sections of different groups stained by H&E stain. Control group **A** showing normal histological structure of mucosa (mu) with (a) normal intact lining epithelium and (g) normal longitudinally arranged glands in lamina propria, underlying submucosa (sub), muscularis (ml), and serosa (S) (scale bar = 100 µm). Ulcer group **B** showing (s) severe epithelial cell sloughing, (n) severe necrosis and distorted arrangement of glands in the mucosa, (f) heavy inflammatory cell infiltration in lamina propria, (h) severe mucosal hemorrhages, and (c) congestion of blood vessels in the mucosal layer with (e) edema and (m) inflammatory cell infiltration in the underlying submucosa (Scale bar = 100 µm). Ulcer group **C** higher magnified stomach section showing (h) a severe hemorrhagic lesion in the mucosal layer and heavy leucocyte infiltration of the lamina propria (arrows) (scale bar = 20 µm). Ulcer group **D** higher magnified stomach section showing (n) necrosed area and (c) congested BVs surrounded by inflammatory cells (arrow) in the mucosal layer. (Scale bar = 20 µm). Pantoprazole group **E** showing (s) moderate sloughing of epithelium, (n) moderate necrosis in lamina propria, (c) congested BVs, and (e) edema with (m) inflammatory cells infiltration in the submucosa. (Scale bar = 100 µm). *Aloe vera* group **F** showing normal histological structure of mucosa with (a) intact lining epithelium, (b) underlying submucosa, (c) muscularis, and (m) few inflammatory cells infiltration. (Scale bar = 100 µm)

Stomach sections of the control group stained with H&E exhibited histologically normal structure with intact lining epithelium and longitudinally distributed glands in the lamina propria, as well as normal submucosa, muscularis, and serosa (Fig. 5A). On the other hand, ulcer group sections revealed severe (+ + +) ulcerative lesions such as severe epithelium destruction, necrosis, and severe hemorrhage in the mucosal layer. Edema, congested BVs, and inflammatory cell infiltration, primarily neutrophils, were seen in the submucosa (Fig. 5B–D). The Pantoprazole stomach sections revealed moderate (+ +) ulcerative alterations compared to the ulcer group. There was moderate epithelial sloughing and necrosis in the mucosal layer, along with moderate submucosal BVs congestion, inflammatory cell infiltration, and moderate edema (Fig. 5E). Whereas *Aloe vera* stomach sections were more similar to those of the control group, as shown by the intact epithelial layer, absence of necrosis and hemorrhage, only mild (+) inflammatory cell infiltration was observed in the submucosa (Fig. 5F). The histopathological changes are shown in Table 4.

**Table 4:** Effect of *Aloe vera* on stomach histopathological changes in ethanol-induced gastric ulcer

Histopathological alteration	Control group	Ulcer group	Pantoprazole group	*Aloe vera* group
Epithelial sloughing in the mucosal layer	−	+++	++	−
Necrosis in the mucosal layer	−	+++	++	−
Hemorrhage in the mucosal layer	−	+++	−	−
inflammatory cell infiltration in the submucosal layer	−	+++	++	+
Edema and BVS congestion in the submucosal layer	−	+++	++	−

− Nil, + Mild, ++ Moderate, +++ Severe

## Discussion

GU is one of the most chronic and recurrent GIT illnesses today. It adversely affects people’s life quality and overall health [28]. Long-term use of anti-secretory medications is costly and causes adverse effects that may outweigh their therapeutic benefits [29]. Therefore, there is a pressing need to discover new, safe, and affordable natural remedies. *Aloe vera* possesses antioxidant, anti-inflammatory, healing, mucus-stimulating, and gastric acid-regulating effects [30]. To the best of our knowledge, this work is the first to assess the effect of *Aloe vera* gel on the NLRP3 and GSDMD signaling pathway as well as the serum gastrin level in GU.

The results demonstrated that *Aloe vera* significantly ameliorated ethanol-induced: (1) acute mucosal injury (decrease UI and provide high I%), (2) oxidative stress (reduced gastric MDA and increased GSH), (3) hypergastrinemia and hyperacidity (decreased serum gastrin and increased gastric pH), and (4) pyroptotic cell death (downregulated gastric NLRP3 and GSDMD mRNA expression). Moreover, histopathological findings supported these results. It’s noteworthy that this study proved that the efficiency of *Aloe vera* for ameliorating ethanol-induced GU was more prominent than that of pantoprazole.

In this research, an ethanol-induced GU model was designed. Due to its higher ulcerogenic effect, ethanol has become the most preferred choice for inducing the experimental ulcer model [31]. Our results revealed that absolute ethanol administered orally induced severe hemorrhagic ulcerative lesions, as quantified by a significant increase in UI. This was confirmed by histopathological investigation, where desquamation of the gastric epithelium, necrosis, acute hemorrhage, submucosal edema, BVs congestion, and inflammatory cells infiltration were observed. Similar results from recent studies have been reported [32, 33]. In contrast, *Aloe vera* treatment significantly lessened gastric damage and improved ulcer healing to a greater extent than the pantoprazole group, as evidenced by the normal intact mucosal surface of the stomach in the *Aloe vera* group, while minor hemorrhagic injuries were visible in the pantoprazole group. This was verified by a significantly decreased UI in the *Aloe vera* group and 91% ulcer inhibition, which is significantly higher than that achieved by pantoprazole (76.17%). These findings were validated by histopathological results where *Aloe vera* effectively repaired the forementioned stomach histopathological alterations triggered by ethanol. Interestingly, *Aloe vera* showed a more potent healing effect than pantoprazole. These findings were consistent with previous studies [6, 34]. *Aloe vera*’s ability to heal wounds may be due to its content of tannins, saponins, and flavonoids as active components. Due to their astringent properties, tannins and other polyphenolic substances have been utilized to treat GU. They react with tissue proteins in the GU, precipitating microproteins at the ulcer site to create a barrier over the damaged tissues. The barrier prevents stomach secretion, protects the underlying mucosa from aggressive stimuli, and accelerates the healing process [35]. Its gel also contains auxin and gibberellin, which act as growth hormones that stimulate cell regeneration and growth to aid in wound healing [36].

Gastric HCL is a crucial factor for the GIT’s ability to absorb iron and calcium, combat infection, and digest food [37]. When it is released in excess, it overcomes the stomach mucosa’s defense mechanisms, leading to GU [38]. Our results showed that gastric pH decreased significantly in the ulcer group. These results agreed with previous studies [39, 40]. On the contrary, treatment with *Aloe vera* increased gastric pH significantly due to inhibition of acid secretion. These findings agreed with previous studies [6, 41]. *Aloe vera*’s inhibitory effect might be because of the existence of lectins in the plant [44]. Lectins prevent parietal cells from absorbing aminopyrine. So, reducing stomach acid is due to a direct impact on acid-secreting cells [42]. When pantoprazole is compared to *Aloe vera*, pantoprazole causes a high rise in gastric pH, which hinders iron and calcium absorption and inhibits the pepsin enzyme, which requires a lower pH to function properly. This resulted in iron deficiency anemia, osteoporosis, and poor food digestion. Also, the absence of stomach acid promoted bacterial growth [43]. *Aloe vera* slightly raises gastric pH, ensuring normal enzyme activities and proper iron and calcium absorption.

Oxidative stress is implicated in the pathophysiology of GU. It develops as a result of an imbalance between ROS and antioxidants that leads to GU. Ethanol induces oxidative stress via lipid peroxidation and the depletion of antioxidants in the stomach mucosa. MDA is the end result of the peroxidation of lipids and is frequently used as a credible indicator of lipid peroxidation [44]. GSH is one of the most powerful antioxidants. Ethanol reduces gastric GSH levels, so it increases ROS accumulation and increases lipid peroxidation as a result [2]. Our findings showed a marked increase in stomach MDA level with a concurrent reduction in stomach GSH level in the ulcer group. These findings were in line with previous studies [45, 46]. As opposed to the ulcer group, *Aloe vera* alleviated oxidative stress, as evidenced by lower stomach MDA and higher gastric GSH levels. These results agreed with previous studies [6, 47]. *Aloe vera*’s powerful antioxidant effect is due to its free radical scavenging activity, reducing ability, and metal chelation [48]. Also, it is due to the high content of antioxidants as enzymes (catalase, superoxide dismutase, and glutathione peroxidase), vitamins (A, E, and C), and phenolic antioxidants (chromones, coumarins, saponins, flavonoids, and tannins). The presence of the OH group prevents oxidative breakdown of the -SH group, conserving tissues’ thiol content and preventing cellular proteins from oxidizing [49].

Gastrin is the primary hormonal inducer for HCL production in both humans and animals. High serum gastrin causes gastric acid hypersecretion, which destroys the gastric mucosa, causing GU [50]. We found a significantly increased serum gastrin level and gastric acid production, confirmed by low gastric pH in the ulcer group. These results concurred with recent studies [40, 51]. The pantoprazole group showed a significantly increased serum gastrin level in comparison with the ulcer group. An increase in gastrin level is a feedback reflex to the hypoacidity (higher gastric pH) induced by the potent acid suppression of PPI. Higher pH inhibits somatostatin release, which increases gastrin release, causing hypergastrinemia [52]. These findings agreed with these studies [53, 54]. Prolonged hypergastrinemia due to chronic PPI use exerts tropic effects in the stomach, which increases the risk of gastric cancer [52]. In contrast, *Aloe vera* significantly decreased serum gastrin level. The exact mechanism through which *Aloe vera* decreased serum gastrin is unclear. Our present findings agree with previous studies that revealed a reduction in serum gastrin level after administration of plant extracts, which have an antiulcer effect [55].

At the molecular level, we examined NLRP3 and GSDMD mRNA expression levels in the gastric mucosa to investigate whether pyroptosis is involved in GU pathophysiology. NLRP3 is a cytoplasmic sensor that is dormant under resting conditions. When it recognizes stimuli as ROS, it is activated, forming the NLRP3 inflammasome. Once the inflammasome is assembled, caspase-1 is activated and induces pro-IL-1β and pro-IL-18 activation. Also, it induces GSDMD cleavage, for triggering pyroptosis and pathological inflammation due to the release of inflammatory cytokines. So, oxidative stress is implicated in pyroptosis [56].

Several studies have demonstrated that decreasing the activity of NLRP3 relieves ethanol-induced GU [57–59]. In accordance with these studies, our study revealed that administration of ethanol significantly upregulated NLRP3 expression, while treatment with *Aloe vera* downregulated its expression and resulted in inhibition of pyroptotic gastric cell death and gastric inflammation. Also, a previous study reported that *Aloe vera* alleviates acute lung injury via inhibition of the NLRP3 inflammatory pathway [60].

GSDMD is a pivotal executioner of pyroptosis. Through its pore-forming action, it induces pyroptosis. Once the NLRP3 inflammasome is assembled, active caspase-1 cleaves GSDMD, which is then translocated to plasma membranes, forming pores that cause cell membrane rupture and pyroptosis [61].Virtually, as the NLRP3 inflammasome was inactivated by *Aloe vera*, caspase-1 activation was decreased. Consequently, the level of cleaved GSDMD was expected to be reduced. Our study exhibited that GSDMD expression is significantly upregulated in the ulcer group. In contrast, *Aloe vera* significantly downregulated its expression. This agreed with recent studies in an ulcerative colitis mouse model [62] and in clinical samples from patients diagnosed with *H. pylori* infection [63].

*Aloe vera* is an immunomodulatory agent that induces an anti-pyroptotic effect. Its significant reduction in GSDMD expression is thought to be a result of decreased NLRP3 expression and NLRP3 inflammasome inactivation. Also, oxidative stress is one of the widely accepted hypotheses for NLRP3 activation, and this effect may be inhibited by ROS scavengers [56]. *Aloe vera* is potent antioxidant and ROS scavenger as mentioned previously. So, it effectively blocks NLRP3 activation signals and reduces pyroptosis. In this respect, we can hypothesize that *Aloe vera*’s therapeutic effects on GU were achieved by inhibiting gastric pyroptotic cell death and inflammation through downregulation of NLRP3 and GSDMD mRNA expression.

## Conclusion

This study revealed that the therapeutic outcome of *Aloe vera* gel on ethanol-induced GU was more effective than pantoprazole. This therapeutic effect of *Aloe vera* could be explained, at least partially, on the basis of the antioxidant (reduction of gastric MDA and elevation of gastric GSH), anti-secretory (decrease serum gastrin and elevate gastric PH), anti-pyroptotic (downregulation NLRP3 and GSDMD mRNA expression), and healing effects (decrease UI, provide high I%, and alleviate histopathological changes). These results recommend *Aloe vera* as a promising phytomedicine that should be evaluated in clinical trials and may be developed as a promising pharmacological agent for GU treatment. Also, this study revealed that pyroptosis is implicated in GU pathophysiology and could offer a novel alternative therapeutic strategy for GU treatment through suppression of pyroptosis.

## Data Availability

Data is available when required.

## Abbreviations

BVs: Blood vessels
ELISA: Enzyme linked immunosorbent assay
GIT: Gastrointestinal tract
GSDMD: Gasdermin D
GSH: Reduced glutathione
GU: Gastric ulcer
I: Inhibition index
MDA: Malondialdehyde
NLRP3: Nucleotide-binding domain, leucine-rich repeat and pyrin domain PYD containing protein 3
qRT-PCR: Quantitative real time-PCR
UI: Ulcer index
